# Identification of Shared Genes and Pathways: A Comparative Study of Multiple Sclerosis Susceptibility, Severity and Response to Interferon Beta Treatment

**DOI:** 10.1371/journal.pone.0057655

**Published:** 2013-02-28

**Authors:** Sunil Mahurkar, Max Moldovan, Vijayaprakash Suppiah, Catherine O’Doherty

**Affiliations:** 1 School of Pharmacy and Medical Sciences, University of South Australia, Adelaide, Australia; 2 Australian Institute of Health Innovation, University of New South Wales, Sydney, Australia; University of Jaén, Spain

## Abstract

Recent genome-wide association studies (GWAS) have successfully identified several gene loci associated with multiple sclerosis (MS) susceptibility, severity or interferon-beta (IFN-ß) response. However, due to the nature of these studies, the functional relevance of these loci is not yet fully understood. We have utilized a systems biology based approach to explore the genetic interactomes of these MS related traits. We hypothesised that genes and pathways associated with the 3 MS related phenotypes might interact collectively to influence the heterogeneity and unpredictable clinical outcomes observed. Individual genetic interactomes for each trait were constructed and compared, followed by prioritization of common interactors based on their frequencies. Pathway enrichment analyses were performed to highlight shared functional pathways. Biologically relevant genes *ABL1, GRB2, INPP5D, KIF1B, PIK3R1, PLCG1, PRKCD, SRC, TUBA1A* and *TUBA4A* were identified as common to all 3 MS phenotypes. We observed that the highest number of first degree interactors were shared between MS susceptibility and MS severity (p = 1.34×10^−79^) with *UBC* as the most prominent first degree interactor for this phenotype pair from the prioritisation analysis. As expected, pairwise comparisons showed that MS susceptibility and severity interactomes shared the highest number of pathways. Pathways from *signalling molecules and interaction*, and *signal transduction* categories were found to be highest shared pathways between 3 phenotypes. Finally, *FYN* was the most common first degree interactor in the MS drugs-gene network. By applying the systems biology based approach, additional significant information can be extracted from GWAS. Results of our interactome analyses are complementary to what is already known in the literature and also highlight some novel interactions which await further experimental validation. Overall, this study illustrates the potential of using a systems biology based approach in an attempt to unravel the biological significance of gene loci identified in large GWAS.

## Introduction

Multiple Sclerosis (MS) is a neurodegenerative disease of the central nervous system (CNS) and is one of the most common CNS diseases in young adults affecting more than 2.5 million people worldwide [1]. MS is a disease of complex aetiology which is believed to be triggered by some yet unconfirmed environmental factors in a genetically predisposed individual. Genome-wide association studies (GWAS) have been particularly successful in identifying genetic variations associated with disease susceptibility [2]–[11]. To date, 11 GWAS and associated meta-analyses have been carried out to determine genetic susceptibility factors for MS. The most recent MS GWAS conducted by the International MS Genetics Consortium analysed the genomes of 9,722 MS patients against 17,376 healthy controls confirming 24 of the previously identified susceptibility loci and discovering an additional 29 novel susceptibility loci [11]. In GWAS studies, stringent statistical significance levels are applied in order to control for false positive associations. However, such stringent statistical significance levels also mean that true associations of modest effects may be discarded [12]. To capture additional information from GWAS data, various computational approaches have been used. For example, Baranzini and colleagues [13] have utilised GWAS data to explore susceptibility pathways relating to MS, while Menon and Farina [14] have attempted to identify shared susceptibility pathways with other autoimmune diseases.

In the current study we have used a similar methodology to Menon and Farina [14] to determine the shared genetic architecture of MS susceptibility, severity and IFN-ß response. We hypothesised that genes/pathways associated with these traits interact collectively to produce the complex and heterogeneous clinical outcome usually seen in MS. On this basis, we proposed that genes/pathways common to two or more phenotypes would be of particular relevance. We thus constructed individual MS susceptibility, severity and IFN-ß response genetic interactomes and identified common interactors in a pairwise fashion. We then prioritised common interactors based on their frequency, and carried out pathway enrichment analyses to highlight functional pathways shared between these traits. Furthermore, we compiled a list of candidate genes likely to interact with or be modulated by MS drugs which have a well understood mechanism of action and aimed to determine common points of interaction in their signalling pathways. Therefore by tackling the question from all angles (GWAS data, functional significance and genes interacting with MS drugs), we aimed to highlight genes which are most likely to be important in modulating the course of MS.

## Materials and Methods

### Genome-wide Association Data

GWAS relating to MS susceptibility (n = 11) [2]–[11], MS severity (n = 2) [4], [15] and IFN-ß response in MS patients (n = 2) [16], [17] were identified using the GWAS Catalog [18] (October 2011 version). This catalog is a comprehensive online resource of association data from published GWAS and related meta-analyses meeting the database inclusion criteria (p-value <1.0×10^−5^). Data relevant to both MS susceptibility and severity was obtained directly from the database. Since the results of the 2 GWAS investigating variable response to IFN-ß therapy in MS patients did not meet the stringent statistical significance threshold in the catalog, no genes were listed in the database. However, for the purpose of this study, we sought to prioritise the genes most likely to be associated with IFN-ß response. As such, genes with p-values less than 0.05 in the validation phase were obtained directly from the 2 pharmacogenomics publications. An overview of the studies is given in Table 1 (Table S1).

**Table 1:** Overview of GWA studies investigated in this analysis.

GWAS Study type	No. of GWAS investigated	GWAS-genes	First degree interactors
MS Susceptibility	11	94	897
MS Severity	2	21	457
Response to IFN-ß treatment	2	19	138

### Interactome Analysis

To investigate the biological interactions and associations of these GWAS-genes from the aforementioned studies, we used VisANT [19], an online tool that allows visualisation, exploration and analysis of gene interactions. It derives interaction data either directly from the literature, or by aligning information from various other interaction databases such as Biogrid, the molecular INTeraction database (MINT), the biomolecular interaction network database (BIND), and Munich information centre for protein sequences (MIPS). Using VisANT, we assembled a list of first degree interactors for GWAS-genes from each category; susceptibility, severity and IFN-ß response (Table S2).

**Figure 1:**
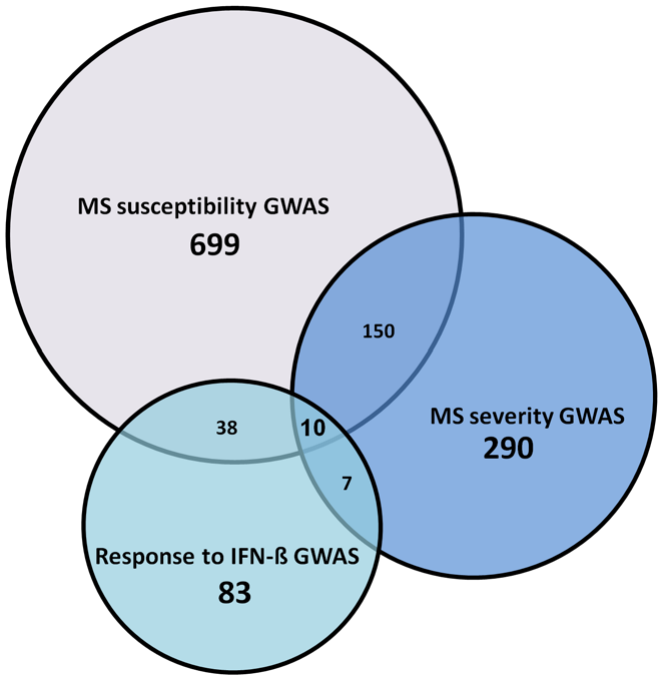
Number of shared first degree interactors between each of the three GWAS phenotype categories.

To investigate the relationships between MS susceptibility, severity and IFN-ß response modifying genes we performed shared comparative interactome analysis between these 3 phenotype categories in a pairwise fashion using methodology similar to Menon and Farina [14] (see Table S3 for details). The statistical significance of the number of shared first degree interactors between any 2 categories was assessed using p-values from a hypergeometric test, which objectively reflect the probability of obtaining the observed or greater number of shared first degree interactors given the common pool of genes under the assumption of no underlying biological mechanism. The test is equivalent to the one-sided Fisher’s test applied to information arranged in a 2×2 contingency table [20] (Table S4). The key entry of the table quantifies the observed numbers of shared first degree interactors between each pair of phenotypes that can be obtained from Figure 1. The corresponding p-value is the probability of obtaining the observed or greater number of first degree interactors belonging to both phenotype 1 and phenotype 2 related sets of first degree interactors, under the null hypothesis of no underlying biological mechanism. Following from this, smaller p-values correspond to stronger evidence against the null hypothesis, thus pointing towards the presence of an underlying biological mechanism.

The paired normalisation factor and interactor score for each phenotype pair were also determined as described previously [14]. Specific to this study, the VisANT interaction ratio and total number of listed interactions for Homo sapiens were 10.64 and 153,297, respectively (October 2011 version). As such, the total number of genes was calculated as 14,407. Details of statistical calculations are given in Table S4.

Further, to identify common interactors of functional relevance, we prioritized these genes based on their frequencies. Details of these common interactors and frequencies of their interaction between any 2 MS related interactomes are given in Table S5.

### Pathway Enrichment Analysis

To elucidate biological networks or pathways most relevant to these common interactors, we performed pathway enrichment analysis using an online tool, ToppGene Suite [21]. ToppGene aids in the identification and prioritization of novel disease candidate genes in the interactome based on functional annotations. For our pathway enrichment analysis, the following default test parameters were used 1) Random sampling size: 1500 (6% of genome); 2) Minimum feature count: 2; 3) Correction: Bonferroni; and 4) Nominal significance level: 5%. Genes which were not identified by ToppGene Suite were removed from further analysis. (For details, see Table S6).

From the list of pathways generated by ToppGene Suite, we classified them based on Kyoto Encyclopedia of Genes and Genomes (KEGG) database’s functional hierarchy schema [22] according to their major functions. Combined p-values were calculated for pathways common between categories using Fisher’s method [23]. Additionally, to identify shared pathways among the 3 MS related Phenotypes, a comparative analysis was performed (Table S7). Reclassification of pathways based on the database was performed to determine the statistical significance and ranks of the overlapping pathways from each distinct database using a hypergeometric test (Table S8).

### The MS Pharmacogenomic Interactome

A search of the literature revealed 7 MS immunomodulatory drugs with reasonably well understood molecular targets/effects. A list of 29 unique candidate genes which encode molecules that directly interact with or are thought to be modulated by these 7 drugs was compiled (Table S9). Using VisANT, we derived a list of first degree interactors for these 29 candidate genes (Table S9) and identified those shared by more than one drug category. In an effort to determine points of convergence between the 29 genes and their interactors (first degree and beyond), we used VisANT to visualise the extended gene-gene interactions. Although IFN-ß is known to bind to the interferon type I receptor, we excluded this drug from this second part of the analysis as we had already prioritised response genes/pathways in the first stage of the study. Interferon is also known to alter the expression of hundreds of genes and this would have ‘drowned out’ data relating to other drugs when processed in VisANT.

## Results

### Constructing the Interactome for MS Susceptibility, MS Severity and IFN-ß Response GWAS

A total of 15 published GWAS within the 3 phenotype categories; MS susceptibility, MS severity and IFN-ß response, were identified from the GWAS catalog. A total of 134 genes were identified as associated with these traits, with some overlap. The MS susceptibility GWAS identified 94 genes – the highest number, whereas studies examining MS severity and IFN-ß response identified 21 and 19 genes, respectively (Table 1). Using VisANT, we then determined the first degree interactors for each of the 3 gene sets. The 94 MS susceptibility genes interacted with a total of 897 first degree interactors, whereas the 21 MS severity and 19 IFN-ß response genes interacted with 457 and 138 first degree interactors, respectively. A list of all genes and their interactors is given in Table S2.

### Pairwise Analysis

Having derived a list of first degree interactors for each category, we then proceeded to determine the extent of overlap between these interactors for each of the 3 MS phenotype categories in a pairwise manner. The results of this analysis indicated that genes associated with susceptibility and severity shared the highest number of interactors, with 160 first degree interactors in common. The susceptibility - IFN-ß response pair shared 48 first degree interactors, while severity - IFN-ß response pair shared only 17 first degree interactors (Figure 1). Of note, 10 genes *ABL1, GRB2, INPP5D, KIF1B, PIK3R1, PLCG1, PRKCD, SRC, TUBA1A* and *TUBA4A* were shared between all 3 GWAS categories (Table S3).

**Figure 2:**
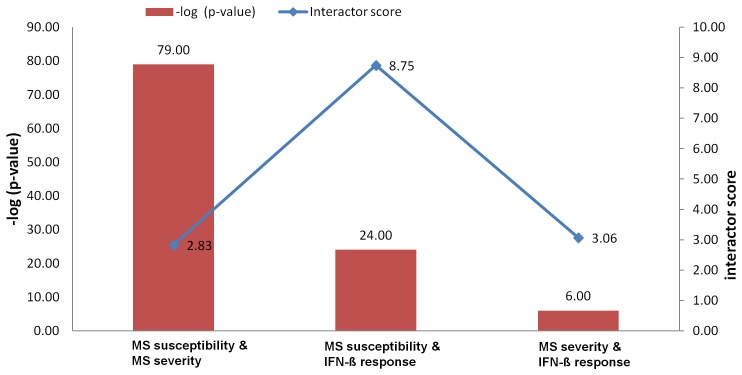
Statistical significance (p-value and interactor score) of shared first degree interactors between paired phenotype categories.

In order to determine the significance of these results, a hypergeometric test was applied to each of the paired categories (Table S4). The susceptibility - severity pair, which had 160 common first degree interacting genes, showed the strongest evidence of statistical significance (p-value = 1.34×10^−79^), followed by the susceptibility - IFN-ß response pair (p-value = 6.89×10^−24^), and then the severity - IFN-ß response pair (p-value = 1.74×10^−6^). Interactor scores were calculated with the intention of eliminating a bias due to varying numbers of studies, generating varying number of interactors and interactions for each gene (Figure 2).

The highest normalized interactor score of 8.75 was obtained for the susceptibility - IFN-ß response pair, indicating a stronger degree of association than might have been perceived based on the raw p-values. The severity - IFN-ß response pair had an interactor score of 3.06, while the susceptibility - severity pair had the lowest score of 2.83.

### Prioritisation of Prominent Individual Interactors Based on Frequency

Given the large number of common interactors for each paired analysis, we attempted to prioritise those most likely to be of functional relevance by determining the frequency with which they interacted with GWAS-genes within each category, and used this to rank them within the paired analyses (Table S5). For example, *UBC*, which codes for ubiquitin C, was a common interactor with 13 susceptibility and 2 severity genes, making it the highest ranked interactor for the susceptibility-severity pair. Phosphatidylinositol 3-kinase regulatory subunit alpha (*PIK3R1)* was ranked second, interacting with 8 susceptibility genes and 2 severity genes. In the susceptibility - IFN-ß response comparison, *GRB2* which codes for growth factor receptor-bound protein 2, was the most commonly encountered gene interacting with 11 susceptibility genes and 2 IFN-ß response genes. *FYN* and *PIK3R1* were next highly ranked for this pair, both interacting with 8 susceptibility genes and 2 IFN-ß response associated genes. Finally, among the severity - IFN-ß response pair, *PIK3R1* was again top ranked along with v-src sarcoma (Schmidt-Ruppin A-2) viral oncogene homolog (*SRC)*. Both of these genes interacted with 2 IFN-ß response genes and 2 severity genes.

### Pathway Enrichment Analysis

In order to investigate the common signalling pathways and the biological functions of the first degree interactors for each gene set, we performed pathway enrichment analysis using ToppGene Suite. Using the default settings, we identified significant pathways enriched by the first degree interactors for each gene set. As ToppGene Suite did not identify some of the genes (Table S6), only 871 interactors from MS susceptibility GWAS, 419 interactors from MS severity GWAS, and 134 interactors from IFN-ß response GWAS were used for further analysis. The results of the ToppGene pathway analysis were derived from 8 different databases namely, KEGG pathway, BioCarta, Reactome, Signalling Gateway, NCI-Nature Curated, PantherDB, Signalling Transduction KE, and Pathway Ontology. The susceptibility interactors were involved in 258 pathways, while the severity and IFN-ß response interactors were involved in 146 and 125 pathways, respectively (Table S6).

**Figure 3:**
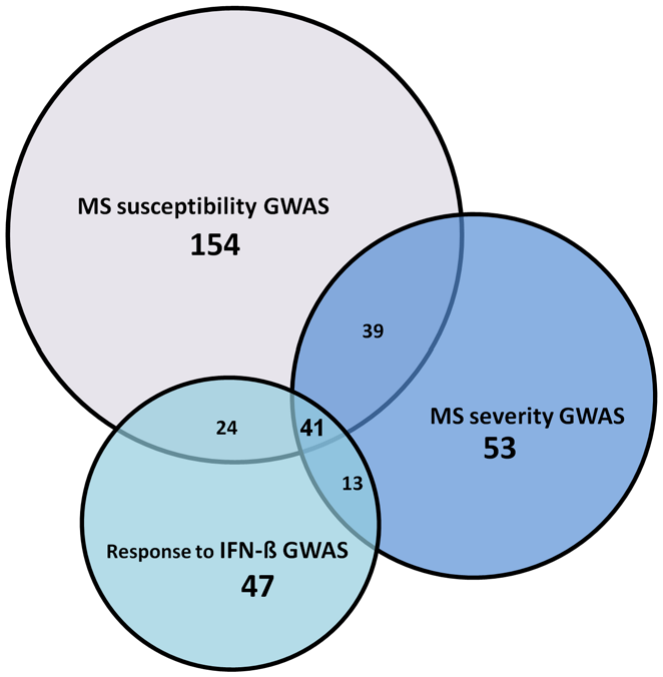
Number of shared pathways relating to the interactomes of the three GWAS phenotype categories.

We then determined pathways shared between the 3 phenotype categories. Pairwise comparisons showed that susceptibility - severity interactomes had the highest number of shared pathways, with a total of 80 pathways common between them, while susceptibility - IFN-ß response interactomes and severity - IFN-ß response interactomes had 65 and 54 common pathways, respectively. Overall, 41 pathways were shared between all 3 phenotype categories (Figure 3). Details of the common pathways and the databases from which they were derived are given in Table S7.

The common pathways were then classified and tabulated according to their functions and source database. Details of the pathways, their biological functions and statistical significance in terms of combined p-values are presented in Table S10. Of the 117 common pathways shared between 2 or more interactomes, a total of 30 pathways had combined p-values of <1.00×10^−16^ and are highlighted in Table S10.

A total of 23 pathways were involved in *Signalling molecules and interaction*. In this class, the most significant shared signalling pathways were those mediated by *CXCR4, stem cell factor receptor (c-Kit), scatter factor/hepatocyte growth factor,* two *IL2* related, and *IL6* pathways. These 6 pathways were shared among all 3 phenotype categories.

In the *Signal transduction* class, 23 statistically significant shared pathways were identified. The 2 most significant among them were the *ErbB* and *Jak-STAT signalling pathways*. The *ErbB signalling pathway* was shared between all 3 phenotype categories, whereas the *Jak-STAT signalling pathway* was shared between the susceptibility and IFN-ß response categories only.

Twenty-two statistically significant common pathways were related to *Cell growth and death*, among which the 9 most significant common pathways were related to signalling mediated by *EGF* (3 databases), *ERbB, PDGFR, FGF*, *hepatocyte growth factor* (2 databases), and also *apoptosis signalling*. These were, for the most part, shared between all 3 phenotype categories with the exception of *apoptosis, FGF,* and *Hepatocyte growth factor receptor* (1 database) *signalling* which were shared by MS susceptibility and MS severity categories.

In the *Immune system* category, 19 pathways were shared between 2 or more phenotype categories. *TCR signalling in naïve CD4+ T cells, T cell activation, FoxO family signalling* and *Fc-epsilon receptor I signalling in mast cells* were among the most statistically significant common pathways between MS susceptibility and severity. The *Toll-like receptor signalling pathway* was highlighted as one of the most significant pathways common to both MS susceptibility and IFN-ß response. Among the 8 pathways that were part of the *Endocrine system*, the *EPO signalling pathway* was the most significant and was shared between all 3 phenotype categories.

There were 5 statistically significant pathways in the *Cancer* category. The most significant of these were *pathways in cancer, colorectal cancer* and *chronic myeloid leukaemia*, which were common to all 3 phenotypes.

Four significant common pathways were identified in each of the following categories: *Infectious diseases*, *Cell communication*, and *Cell development*. In the *Infectious diseases* class, *NFkB activation by nontypeable Hemophilus influenzae* was the most significant pathway (p-value = 3.33×10^−16^) and was shared amongst susceptibility and IFN-ß response categories. Signalling *events mediated by PTP1B* was the most significant pathway in the *Cell communication* class which was shared between susceptibility and IFN-ß response categories. In the *Development* category, there were 2 particularly notable pathways. *Keratinocyte differentiation* was common between the susceptibility and severity categories, while the *Angiogenesis* pathway was shared between the severity and IFN-ß response categories.

Three statistically significant common pathways were related to the *Nervous system.* Of these, the most significant was *Genes involved in signalling by NGF,* which was shared amongst all 3 phenotype categories. Finally, 2 shared pathways were classified under *Neurodegenerative diseases.* The *Parkinson’s disease* pathway, which was shared amongst MS susceptibility and severity categories, was the most significant (p-value = 2.06×10^−7^).

In order to quantify the significance of the pathway overlap, we again performed a pairwise analysis of the phenotype categories using a hypergeometric test. The results of this analysis, performed using the data for each of the 8 individual databases, are tabulated in Table S8. The overlaps were ranked from 1 through 3 based on corresponding p-values.

The severity - IFN-ß response pair had the most significant pathway overlaps for KEGG, Reactome and NCI-Nature Curated databases (p-values of 4.82×10^−8^, 3.86×10^−4^ and 1.59×10^−5^ respectively). Interestingly, only BioCarta and PantherDB databases ranked the pathways common between susceptibility and severity as most significant (p-values of 2.70×10^−8^ and 1.28×10^−5^ respectively). Pathway Ontology database ranked the susceptibility - IFN-ß response pair as most significant in terms of pathway overlaps (p-values of 2.37×10^−6^).

### Convergent Pathways of MS Drugs

We were also interested to determine if targeted MS immunomodulatory treatments with well-defined molecular interactions might have common interactors in their signalling networks. Such common interactors might be putative novel drug targets. A literature search was conducted to determine, firstly, drugs with defined targets, and secondly, a list of protein molecules thought to directly interact with or be modulated by each drug. In total, 7 drugs were identified, namely alemtuzumab, cladribine, fingolimod, mitoxantrone, natalizumab, ocrelizumab and teriflunomide which are either in development, licensed or were previously licensed but taken off label. In some instances, one drug was a representative of a class of drugs with the same molecular target (ocrelizumab representing the CD20 antagonists). The list of drugs and interacting proteins is provided in Table S9.

We then used VisANT to determine the first degree interactors for the genes encoding these 29 molecules. Five genes (*CD52, NT5C1A, SLC28A2, SLC28A* and *SLC29A2*) did not have any first degree interactors and were excluded from further analysis. Of the remaining 24 genes, we then determined first degree interactors which were common between multiple drugs. There were 26 first degree interactors associated with 2 or more of the aforementioned 7 MS drugs. Details of the VisANT output and drug sets with common interactors are given in Table S9. Of note, the most common shared first degree interactor, *FYN* interacts with pathways modulated by 4 drugs, alemtuzumab and cladribine (*CASP3*), fingolimod (*SPHK1* and *SPHK2*) and ocrelizumab (*MS4A1*).

**Figure 4:**
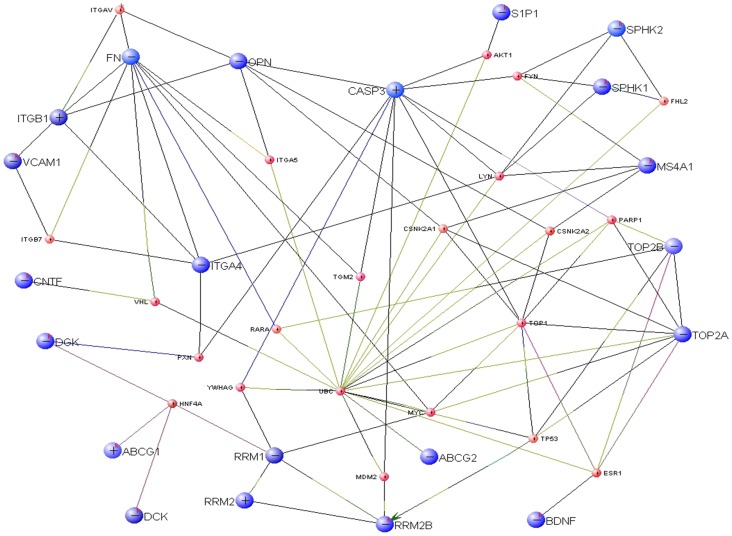
Primary “drug modulated/modulating” genes (large blue circles) and their extended common interactors (small red circles). ‘+’ on each gene (node) indicates that the gene’s linkages are suppressed and ‘−’ indicates all linkages of the gene have been shown. The different coloured interaction between genes represents various biological processes that identified the interactions. Every line (edge) connecting each gene pair represents an interaction. The colour of the line specifies the experimental method used to identify that interaction. For example, “black” coloured line connecting genes *ITGAV* and *FN* represents the *transcriptional upregulation* method. If an interaction is identified by several different biological methods, then the line will be coloured in segments with corresponding colours for each methods. For example 5 different colours indicated in line connecting genes *ITGAV* and *ITGB1* represent 5 biological methods used for identification of this interaction i.e. *in vivo, inferred by curator, affinity chromatography, co- immunoprecipitation* and *pull down* method.

We then used VisANT to visualise the points of convergence in the extended drug modulating/modulated pathways, i.e. beyond first degree interactors. Figure 4 illustrates the complex network of these gene-gene interactions with the primary drug modulating/modulated genes (large blue circles) on the periphery and common interactors (small red circles) in the centre.

*UBC*, which is involved in many critical processes, such as activation of protein kinases and signalling, was identified as the greatest point of convergence within this network with 18 interactions. It is a first degree interactor of primary drug modulating/modulated genes associated with mitoxantrone *(TOP2A, ABCG2)* and teriflunomide *(ABCG2),* and is additionally a second degree interactor of genes associated with alemtuzumab *(CASP3, CNTF* and *BDNF),* cladribine *(CASP3, RRM1* and *RRMB2)* ocrelizumab (*MS4A1*), fingolimod (*S1P1*, *SPHK1* and SPHK2), mitoxantrone (*TOP2B*) and natalizumab (*ITGA4*, *FN* and *OPN*).

In the interaction network, *CASP3* which had a total of 10 interactions, was observed to be one of the primary drug modulated/modulating genes, in relation to both alemtuzumab and cladribine. Of particular interest, it is also a second degree interactor of the actual molecular targets of fingolimod *(S1P)*, mitoxantrone (*TOP2B, TOP2A*), natalizumab (*ITGA4*) and ocrelizumab (*MS4A1*).

## Discussion

Systems biology based approaches exploring genetic pathways and networks offer insights into the pathogenesis of complex diseases at the cellular level and may aid in the identification of novel drug targets. Whereas shared GWAS genes such as *GPC5,* which is both a susceptibility [4], [24] and response modifying [16], [25] gene, is relatively obvious, we were more interested in the extended interaction between networks related to various MS traits. In this study using the systems biology based approach, we utilised published GWAS data investigating 3 MS related phenotypes - susceptibility, severity and pharmacogenomic IFN-ß response - to explore and mine common interactomes and functional pathways between the 3 phenotypes. Following this we explored interactions between candidate genes, either affected by or effectors of MS medications and how these interactions fit within the underlying GWAS data.

Results of the pairwise analysis indicated a substantial overlap of the MS susceptibility, severity and IFN-ß response interactomes. The raw data suggested greatest sharing between the susceptibility - severity pair, and least between the severity - IFN-ß response pair, and this trend was later observed for the pathway analysis. This is somewhat distorted by the large number of susceptibility studies relative to severity and IFN-ß response studies (susceptibility, n = 11; severity, n = 2; IFN-ß response, n = 2). By calculating normalised interactor scores we were able to show that, based on the evidence available to date, the greatest degree of sharing actually occurs between the susceptibility and IFN-ß response interactomes. This finding should be interpreted with caution until additional evidence better covering the latter two phenotypes becomes available. We identified 10 first degree interactors common to all 3 phenotypes. The overlap of these first degree interactors highlights the importance of genetic interaction among genes and their pathways influencing determinants of MS susceptibility, disease severity and IFN-ß response. Indeed, the biological functions, cellular pathways and regulatory mechanisms of the proteins encoded by these genes in the nervous and immune systems have been previously documented. It has been demonstrated that mice deficient of ABL1 have impaired development and responsiveness of T cells and B cells [26], [27]. SHIP-1 encoded by *INPP5D* was found to be critical for normal Th17 cell development and known to play a key role in the reciprocal regulation of regulatory T cells and Th17 cells [28]. *INPP5D*, involved in signal transduction was found to be significantly upregulated as a consequence of disease development in EAE mice, the animal model of MS when compared to healthy mice (mean-fold change = 15.0, p-value <0.01) [29]. Additionally, in an *in silico* study of MS lesion-specific proteins, *INPP5D* was suggested to be a part of gene network associated with chronic active MS plaques [30]. A member of the kinesin superfamily, Kif1b, is critical for intracellular transport, an essential process for neuronal morphogenesis, function and survival. Kif1b is also required in localization of myelin mRNA to oligodendrocyte processes for myelin biogenesis [31]. Protein kinase C delta (PRKCD) is an oxidative stress-sensitive kinase which functions as a key mediator in apoptotic cell death in various cell types including neuronal cells [32]. Thus, PRKCD’s role in mediation and promotion of apoptotic cell death is considered to be critical in the pathogenesis of neurodegenerative disorders, such as Parkinson’s disease [33].

Proteins encoded by *GRB2, PIK3R1, PLCG1* are key players in a major signal-transduction pathway mediated by brain-derived neurotrophic factor (BDNF). Genetic analyses of polymorphisms in the *BDNF* gene region have generated inconclusive associations in terms of both MS susceptibility and severity [34]–[37]. Several studies have evaluated the possible role of IFN-ß in modulating BDNF production in MS patients. Higher levels of BDNF have been observed in peripheral blood mononuclear cells (PBMCs) of IFN-ß-treated MS patients compared to non-treated patients [38], [39]. Additionally, with glatiramer acetate (GA) treatment, higher levels of BDNF were observed only in responders. It was suggested that these elevated levels of BDNF in GA responders were associated with regulation of peripheral T cells [40], [41]. The biological functions of the genes identified by our computational analyses seem to substantiate the potential relevance of our findings.

Prioritisation of common genes in the paired analysis based on their frequency again identified *PIK3R1* which was the only first degree interactor shared among all 3 phenotypes. *UBC,* which had a total of 15 connections with MS susceptibility and severity genes, was the most well connected gene in the MS drugs-gene network (see further discussion below). *UBC* is known to be involved in various critical processes in the CNS [42]. *FYN* was specifically found to be a frequent and unique interactor in the susceptibility - IFN-ß response pair. Fyn, a member of the Src kinase family, is known to be a key regulator of multiple downstream signalling pathways leading to differentiation of oligodendrocytes, the myelinating cells of the CNS [43]–[45]. Fyn kinase is also found to play an important role in the production and regulation of cytokines [46] and is known to have a significant role in the immune system modulating the balance between highly specialized Th17 cell and regulatory T (Treg) cells [47]. It is now well established that, autoimmune diseases, including MS, are associated with increased levels of Th17 cells and decreased levels of Treg cells [48].

Pathway analysis of the first degree interactors indicated 41 pathways common to 2 or more phenotypes, suggesting a plausible link between these different aspects of the disease. Interestingly, most of the statistically noticeable common pathways identified by the pairwise analysis (Table S6) activate the following critical signal transduction pathways: the MAPKs pathways, PI3-k signalling pathway, JAK-STAT signalling pathway, PLC-Gamma and SRC pathways. Not surprisingly, JAK-STAT signalling was one of the most significantly shared pathways between susceptibility and IFN-ß response. It was also of interest that IL-7 signal transduction was highlighted for this phenotype pair since IL-7 production has been implicated in functional studies as a determinant of IFN-ß response [49].

With our group’s specific interest in pharmacogenomics, we were particularly keen to identify the most likely genes/pathways involved in IFN-ß response and other MS treatments. By finding common first degree interactors and points of convergence in the extended networks of treatments with well understood mechanisms of action, we hoped to identify commonalties which may be potential drug targets. *FYN* was the most common first degree interactor in the drug network. This was particularly interesting since *FYN* was also a frequent and unique first degree interactor between IFN-ß response and susceptibility in the first stage of the analysis. Furthermore, given the functional relevance of this protein (as described above) we believe *FYN* is a good candidate for further investigation as a modulator of disease activity in MS. Some other functionally interesting but less common first degree interactors included paxillin (*PXN*) which is believed to play a critical role in oligodendrocyte differentiation and ultimately regulation of myelination [50], AKT1 which also plays a role in processes such as regulation of neuronal differentiation and survival [51], and Lyn which has immunoregulatory functions [52]. *LYN* was also a common interactor between susceptibility and IFN-ß response interactomes. *MYC* was a common first degree interactor for 3 drugs (cladribine, mitoxantrone, natalizumab) and was noted to be an interactor in the susceptibility- IFN-ß response paired analysis. A subset of common first degree interactors of drugs were also highlighted in the shared susceptibility- severity analysis: *SPP1*, *PRKDC*, *HSPD1*, *YWHAE*, *CTNNB1*, *ESR1*, *TP53* and *UBC*.

In the convergent pathways analysis of MS drugs, *UBC* and *CASP3* were the 2 genes with the most number of connections. Ubiquitin, a highly conserved protein, is known to play a critical role as a signalling molecule in various proteolytic and non-proteolytic pathways. Along with UBB, UBA52 and UBA80, UBC maintains the ubiquitin homeostasis, a critical factor in the ubiquitin-proteasome system (UPS). UPS is responsible for the degradation of irreversibly damaged and misfolded proteins. Along with this critical housekeeping role, UPS is also responsible for regulation of various protein functions via ubiquitination [53]. In a recent study, significantly elevated levels of plasma ubiquitin and proteasome enzymatic activities (encompassing of chymotrypsin-like (Ch-L), trypsin-like (Tr-L) and caspase-like activity) were found in pre IFN-ß treated MS patients compared to healthy controls (p-value <0.01). Also, the UPS enzymatic activities were significantly higher pre IFN-ß treatment in these patients (p-value <0.0001) compared to post treatment. The authors proposed that the inhibition of the UPS system through IFN-ß therapy improved the clinical course of MS [54]. However, we did not identify *UBC* as a common first degree interactor in our analysis of the IFN-ß interactome with susceptibility and/or severity interactomes.

The pharmacological actions of both alemtuzumab and cladribine seem to involve the activation of CASP-3, a member of the cysteine-aspartic acid protease (caspase) family which is the second most connected gene in the convergent pathways analysis. Sequential activation of caspase proteins regulates various critical functions including cell survival, proliferation, differentiation and particularly apoptosis [55]. Uncontrolled cell proliferation or apoptosis have been implicated in the pathogenesis of various cancers and autoimmune diseases such as systemic lupus erythematosus [56]. Apoptotic loss of neurons has been reported in cortical MS lesions and in rat model of MS [57], [58]. O’Doherty and colleagues analysed 61 relevant SNPs in 155 responding and 100 non-responding Irish MS patients and found a *JAK2–IL10–CASP3* allelic combination to be predictive of response status (p-value = 4.0×10^−4^) [59]. In the first stage of our analysis, *CASP3* was a common interactor in the severity- IFN-ß response pair but not in the susceptibility- IFN-ß response analysis.

The aim of this study was to primarily determine the shared genes/pathways between 3 MS related characteristics: susceptibility, severity, and IFN-ß response. We hypothesised that these traits must intertwine at a biological level, and thus those overlapping genes/pathways would be the most functionally relevant. To date, only the data from susceptibility studies has been sufficiently powered and extensively validated, and this is a major limitation of our analysis. However, we believe that by using this robust susceptibility data our study has been able to tease out the most likely genes/pathways influencing severity and/or IFN-ß response. A further caveat of this study is that current IFN- ß response criteria may incidentally capture patients with a less/more aggressive disease course. On this basis, it is difficult to determine whether shared genes/pathways in the response/severity interactomes are indeed common, or if they are all MS severity related genes/pathways with other distinct IFN- ß interactors representing true response modulators. Additionally, results related to this phenotype pair are not underpinned by the adequately powered/validated susceptibility data.

Our investigation of MS related GWAS-genes, their first degree interactors and related functional pathways led to the identification of genes and pathways that are highly plausible candidates as MS biomarkers and/or therapeutic targets. We identified 10 interactors common to all three MS phenotypes – *ABL1*, *GRB2*, *INPP5D*, *KIF1B*, *PIK3R1*, *PLCG1*, *PRKCD*, *SRC*, *TUBA1A* and *TUBA4A*, and identified a number of common genes in drug signalling pathways. *FYN*, in particular, is an appealing candidate for further investigation due to its frequency in our drugs network, presence in the susceptibility - IFN-ß response shared interactome, and its functional relevance in MS. Thus, by utilizing the wealth of information generated by GWAS and employing such complementary systems-based analyses, it is plausible to identify the underlying biological themes driving the various disease traits in a complex disease like MS. Appropriate follow-up studies are needed to further investigate and validate the role of the genes and pathways identified in this study with respect to various disease processes in MS.

## Supporting Information

Table S1 **Detailed records of GWAS from the GWAS catalog used in these analyses.**
(XLSX)

Table S2 **Interactome details – List of 3 MS related GWAS-genes and their interactors.**
(XLS)

Table S3 **List of the shared interactors from all three GWAS categories.**
(XLSX)

Table S4 **Statistical significances for interactor sharing – calculations of VisANT interactor score and VisANT interaction ratio.**
(XLS)

Table S5 **Frequency of common interactors.**
(XLS)

Table S6 **Details of pathway enrichment analysis – input data and results.**
(XLS)

Table S7 **List of shared pathways between 3 MS related phenotypes.**
(XLS)

Table S8 **Details of statistical significances and ranking of overlapping pathways between 3 MS related phenotypes.**
(XLS)

Table S9 **List of MS drugs and their possible mechanism of action along with gene/s involved. Interactome analysis of MS drugs-genes.**
(XLS)

Table S10 **Functional classification of common pathways between three phenotype categories based on the database from which they were derived.**
(XLS)

## References

[1] Alonso A, Hernán MA (2008) Temporal trends in the incidence of multiple sclerosis: A systematic review. Neurology 71: 129–135. 18606967 10.1212/01.wnl.0000316802.35974.34 PMC4109189

[2] International Multiple Sclerosis Genetics Consortium, Hafler DA, Compston A, Sawcer S, Lander ES, et al (2007) Risk alleles for multiple sclerosis identified by a genomewide study. N Engl J Med 357: 851–862. 17660530 10.1056/NEJMoa073493

[3] Aulchenko YS, Hoppenbrouwers IA, Ramagopalan SV, Broer L, Jafari N, et al (2008) Genetic variation in the KIF1B locus influences susceptibility to multiple sclerosis. Nat Genet 40: 1402–1403. 18997785 10.1038/ng.251

[4] Baranzini SE, Wang J, Gibson RA, Galwey N, Naegelin Y, et al (2009) Genome-wide association analysis of susceptibility and clinical phenotype in multiple sclerosis. Hum Mol Genet 18: 767–778. 19010793 10.1093/hmg/ddn388 PMC4334814

[5] De Jager PL, Jia X, Wang J, de Bakker PI, Ottoboni L, et al (2009) Meta-analysis of genome scans and replication identify CD6, IRF8 and TNFRSF1A as new multiple sclerosis susceptibility loci. Nat Genet 41: 776–782. 19525953 10.1038/ng.401 PMC2757648

[6] The Australia and New Zealand Multiple Sclerosis Genetics Consortium (ANZgene), Bahlo M, Booth DR, Broadley SA, Brown MA, Footeet SJ, et al (2009) Genome-wide association study identifies new multiple sclerosis susceptibility loci on chromosomes 12 and 20. Nat Genet 41: 824–828. 19525955 10.1038/ng.396

[7] Jakkula E, Leppä V, Sulonen AM, Varilo T, Kallio S, et al (2010) Genome-wide association study in a high-risk isolate for multiple sclerosis reveals associated variants in STAT3 gene. Am J Hum Genet 86: 285–291. 20159113 10.1016/j.ajhg.2010.01.017 PMC2820168

[8] Nischwitz S, Cepok S, Kroner A, Wolf C, Knop M, et al (2010) Evidence for VAV2 and ZNF433 as susceptibility genes for multiple sclerosis. J Neuroimmunol 227: 162–166. 20598377 10.1016/j.jneuroim.2010.06.003

[9] Baranzini SE, Srinivasan R, Khankhanian P, Okuda DT, Nelson SJ, et al (2010) Genetic variation influences glutamate concentrations in brains of patients with multiple sclerosis. Brain 133: 2603–2611. 20802204 10.1093/brain/awq192 PMC2929334

[10] Wang JH, Pappas D, De Jager PL, Pelletier D, de Bakker PI, et al (2011) Modeling the cumulative genetic risk for multiple sclerosis from genome-wide association data. Genome Med 3: 3. 21244703 10.1186/gm217 PMC3092088

[11] The International Multiple Sclerosis Genetics Consortium & The Wellcome Trust Case Control Consortium 2, Sawcer S, Hellenthal G, Pirinen M, Spencer C, Nikolaos A et al (2011) Genetic risk and a primary role for cell-mediated immune mechanisms in multiple sclerosis. Nature 476: 214–219. 21833088 10.1038/nature10251 PMC3182531

[12] Altshuler D, Hirschhorn JN, Klannemark M, Lindgren CM, Vohl MC, et al (2000) The common PPAR[gamma] Pro12Ala polymorphism is associated with decreased risk of type 2 diabetes. Nat Genet 26: 76–80. 10973253 10.1038/79216

[13] Baranzini SE, Galwey NW, Wang J, Khankhanian P, Lindberg R, et al (2009) Pathway and network-based analysis of genome-wide association studies in multiple sclerosis. Hum Mol Genet 18: 2078–2090. 19286671 10.1093/hmg/ddp120 PMC2678928

[14] Menon R, Farina C (2011) Shared molecular and functional frameworks among five complex human disorders: A comparative study on interactomes linked to susceptibility Genes. PLoS ONE 6: e18660. 21533026 10.1371/journal.pone.0018660 PMC3080867

[15] International Multiple Sclerosis Genetics Consortium, Briggs FB, Shao X, Goldstein BA, Oksenberg JR, Barcellos LF, et al (2011) Genome-wide association study of severity in multiple sclerosis. Genes Immun 12: 615–625. 21654844 10.1038/gene.2011.34 PMC3640650

[16] Byun E, Caillier SJ, Montalban X, Villoslada P, Fernández O, et al (2008) Genome-wide pharmacogenomic analysis of the response to interferon beta therapy in multiple sclerosis. Arch Neurol 65: 337–344. 18195134 10.1001/archneurol.2008.47

[17] Comabella M, Craig DW, Morcillo-Suárez C, Río J, Navarro A, et al (2009) Genome-wide scan of 500 000 single-nucleotide polymorphisms among responders and nonresponders to interferon beta therapy in multiple sclerosis. Arch Neurol 66: 972–978. 19667218 10.1001/archneurol.2009.150

[18] Hindorff LA, Sethupathy P, Junkins HA, Ramos EM, Mehta JP, et al (2009) Potential etiologic and functional implications of genome-wide association loci for human diseases and traits. Proc Natl Acad Sci U S A 106: 9362–9367. 19474294 10.1073/pnas.0903103106 PMC2687147

[19] Hu Z, Ng DM, Yamada T, Chen C, Kawashima S, et al (2007) VisANT 3.0: new modules for pathway visualization, editing, prediction and construction. Nucleic Acids Res 35: W625–W632. 17586824 10.1093/nar/gkm295 PMC1933155

[20] Rivals I, Personnaz L, Taing L, Potier MC (2007) Enrichment or depletion of a GO category within a class of genes: which test? Bioinformatics 23: 401–407. 17182697 10.1093/bioinformatics/btl633

[21] Chen J, Aronow BJ, Jegga AG (2009) Disease candidate gene identification and prioritization using protein interaction networks. BMC Bioinformatics 10: 73. 19245720 10.1186/1471-2105-10-73 PMC2657789

[22] Kanehisa M, Goto S, Sato Y, Furumichi M, Tanabe M (2012) KEGG for integration and interpretation of large-scale molecular data sets. Nucleic Acids Res 40: D109–D114. 22080510 10.1093/nar/gkr988 PMC3245020

[23] Mosteller CF, Fisher RA (1948) Questions and answers: Combining independent tests of significance. Am Stat 2: 30–31.

[24] Cavanillas ML, Fernández O, Comabella M, Alcina A, Fedetz M, et al (2011) Replication of top markers of a genome-wide association study in multiple sclerosis in Spain. Genes Immun 12: 110–115. 20944657 10.1038/gene.2010.52

[25] Cénit MD, Blanco-Kelly F, de las Heras V, Bartolomé M, de la Concha EG, et al (2009) Glypican 5 is an interferon-beta response gene: a replication study. Mult Scler 15: 913–917. 19556317 10.1177/1352458509106509

[26] Silberman I, Sionov RV, Zuckerman V, Haupt S, Goldberg Z, et al (2008) T cell survival and function requires the c-Abl tyrosine kinase. Cell Cycle 7: 3847–3857. 19098427 10.4161/cc.7.24.7267 PMC3407662

[27] Brightbill H, Schlissel MS (2009) The effects of c-Abl mutation on developing B cell differentiation and survival. Int Immunol 21: 575–585. 19299624 10.1093/intimm/dxp027 PMC2675032

[28] Locke NR, Patterson SJ, Hamilton MJ, Sly LM, Krystal G, et al (2009) SHIP regulates the reciprocal development of T regulatory and Th17 cells. J Immunol 183: 975–983. 19542365 10.4049/jimmunol.0803749

[29] Matejuk A, Hopke C, Dwyer J, Subramanian S, Jones RE, et al (2003) CNS gene expression pattern associated with spontaneous experimental autoimmune encephalomyelitis. J Neurosci Res 73: 667–678. 12929134 10.1002/jnr.10689

[30] Satoh JI, Tabunoki H, Yamamura T (2009) Molecular network of the comprehensive multiple sclerosis brain-lesion proteome. Mult Scler 15: 531–541. 19389748 10.1177/1352458508101943

[31] Lyons DA, Naylor SG, Scholze A, Talbot WS (2009) Kif1b is essential for mRNA localization in oligodendrocytes and development of myelinated axons. Nat Genet 41: 854–858. 19503091 10.1038/ng.376 PMC2702462

[32] Jin H, Kanthasamy A, Anantharam V, Rana A, Kanthasamy AG (2011) Transcriptional regulation of pro-apoptotic protein kinase Cδ. J Biol Chem 286: 19840–19859. 21467032 10.1074/jbc.M110.203687 PMC3103361

[33] Kaul S, Kanthasamy A, Kitazawa M, Anantharam V, Kanthasamy AG (2003) Caspase-3 dependent proteolytic activation of protein kinase Cδ mediates and regulates 1-methyl-4-phenylpyridinium (MPP+)-induced apoptotic cell death in dopaminergic cells: Relevance to oxidative stress in dopaminergic degeneration. Eur J Neurosci 18: 1387–1401. 14511319 10.1046/j.1460-9568.2003.02864.x

[34] Mero IL, Smestad C, Lie BA, Lorentzen ÅR, Sandvik L, et al (2012) Polymorphisms of the BDNF gene show neither association with multiple sclerosis susceptibility nor clinical course. J Neuroimmunol 244: 107–110. 22341604 10.1016/j.jneuroim.2012.01.011

[35] Blanco Y, Gómez-Choco M, Arostegui JL, Casanova B, Martínez-Rodríguez JE, et al (2006) No association of the Val66Met polymorphism of brain-derived neurotrophic factor (BDNF) to multiple sclerosis. Neurosci Lett 396: 217–219. 16356643 10.1016/j.neulet.2005.11.032

[36] Lindquist S, Schott BH, Ban M, Compston DA, Sawcer S, et al (2005) The BDNF-Val66Met polymorphism: Implications for susceptibility to multiple sclerosis and severity of disease. J Neuroimmunol 167: 183–185. 16046000 10.1016/j.jneuroim.2005.06.008

[37] Mirowska-Guzel D, Mach A, Gromadzka G, Czlonkowski A, Czlonkowska A (2008) BDNF A196G and C270T gene polymorphisms and susceptibility to multiple sclerosis in the polish population. Gender differences. J Neuroimmunol 193: 170–172. 18061279 10.1016/j.jneuroim.2007.10.013

[38] Lalive PH, Kantengwa S, Benkhoucha M, Juillard C, Chofflon M (2008) Interferon-ß induces brain-derived neurotrophic factor in peripheral blood mononuclear cells of multiple sclerosis patients. J Neuroimmunol 197: 147–151. 18555540 10.1016/j.jneuroim.2008.04.033

[39] Yoshimura S, Ochi H, Isobe N, Matsushita T, Motomura K, et al (2010) Altered production of brain-derived neurotrophic factor by peripheral blood immune cells in multiple sclerosis. Mult Scler 16: 1178–1188. 20656764 10.1177/1352458510375706

[40] Blanco Y, Moral EA, Costa M, Gómez-Choco M, Torres-Peraza JF, et al (2006) Effect of glatiramer acetate (Copaxone®) on the immunophenotypic and cytokine profile and BDNF production in multiple sclerosis: A longitudinal study. Neurosci Lett 406: 270–275. 16934924 10.1016/j.neulet.2006.07.043

[41] Blanchette F, Neuhaus O (2008) Glatiramer Acetate. J Neurol 255: 26–36. 18317674 10.1007/s00415-008-1005-5

[42] Mabb AM, Ehlers MD (2010) Ubiquitination in Postsynaptic Function and Plasticity. Annu Rev Cell Dev Biol 26: 179–210. 20604708 10.1146/annurev-cellbio-100109-104129 PMC3163670

[43] Seiwa C, Yamamoto M, Tanaka K, Fukutake M, Ueki T, et al (2007) Restoration of FcRγ/Fyn signaling repairs central nervous system demyelination. J Neurosci Res 85: 954–966. 17290413 10.1002/jnr.21196

[44] Laursen LS, Chan CW, Constant CF (2009) An integrin-contactin complex regulates CNS myelination by differential Fyn phosphorylation. J Neurosci 29: 9174–9185. 19625508 10.1523/JNEUROSCI.5942-08.2009 PMC4017644

[45] Krämer-Albers EM, White R (2011) From axon–glial signalling to myelination: The integrating role of oligodendroglial Fyn kinase. Cell Mol Life Sci 68: 2003–2012. 21207100 10.1007/s00018-010-0616-z PMC11114493

[46] Gomez G, Gonzalez-Espinosa C, Odom S, Baez G, Cid ME, et al (2005) Impaired FcεRI-dependent gene expression and defective eicosanoid and cytokine production as a consequence of Fyn deficiency in mast cells. J. Immunol 175: 7602–7610. 16301670 10.4049/jimmunol.175.11.7602

[47] Ueda A, Zhou L, Stein PL (2012) Fyn Promotes Th17 Differentiation by Regulating the Kinetics of RORγt and Foxp3 Expression. J Immunol 188: 5247–5256. 22539787 10.4049/jimmunol.1102241 PMC3358535

[48] Jäger A, Kuchroo VK (2010) Effector and regulatory T-cell subsets in autoimmunity and tissue inflammation. Scand J Immunol 72: 173–184. 20696013 10.1111/j.1365-3083.2010.02432.x PMC3129000

[49] Lee LF, Axtell R, Tu GH, Logronio K, Dilley J, et al (2011) IL-7 promotes T(H)1 development and serum IL-7 predicts clinical response to interferon-β in multiple sclerosis. Sci Transl Med 3: 93ra68. 10.1126/scitranslmed.3002400 PMC3739690 21795588

[50] Miyamoto Y, Yamauchi J, Chan JR, Okada A, Tomooka Y, et al (2007) Cdk5 regulates differentiation of oligodendrocyte precursor cells through the direct phosphorylation of paxillin. J Cell Sci 120: 4355–4366. 18042622 10.1242/jcs.018218

[51] Vojtek AB, Taylor J, DeRuiter SL, Yu JY, Figueroa C, et al (2003) Akt regulates basic helix-loop-helix transcription factor-coactivator complex formation and activity during neuronal differentiation. Mol Cell Biol. 23: 4417–4427. 10.1128/MCB.23.13.4417-4427.2003 PMC164860 12808085

[52] Ingley E (2012) Functions of the Lyn tyrosine kinase in health and disease. Cell Commun Signal 10: 21. 22805580 10.1186/1478-811X-10-21 PMC3464935

[53] Baptista MS, Duarte CB, Maciel P (2012) Role of the ubiquitin–proteasome system in nervous system function and disease: using C. elegans as a dissecting tool. Cell Mol Life Sci 69: 2691–2715. 22382927 10.1007/s00018-012-0946-0 PMC11115168

[54] Minagar A, Ma W, Zhang X, Wang X, Zhang K, et al (2012) Plasma ubiquitin-proteasome system profile in patients with multiple sclerosis: correlation with clinical features, neuroimaging, and treatment with interferon-beta-1b. Neurol Res 34: 611–618. 22709658 10.1179/1743132812Y.0000000055

[55] Lamkanfi M, Festjens N, Declercq W, Vanden Berghe T, Vandenabeele P (2007) Caspases in cell survival, proliferation and differentiation. Cell Death Differ 14: 44–55. 17053807 10.1038/sj.cdd.4402047

[56] Lorenz HM, Herrmann M, Winkler T, Gaipl U, Kalden JR (2000) Role of apoptosis in autoimmunity. Apoptosis 5: 443–449. 11256887 10.1023/a:1009692902805

[57] Peterson JW, Bö L, Mörk S, Chang A, Trapp BD (2001) Transected neurites, apoptotic neurons, and reduced inflammation in cortical multiple sclerosis lesions. Ann Neurol 50: 389–400. 11558796 10.1002/ana.1123

[58] Meyer R, Weissert R, Diem R, Storch MK, de Graaf KL, et al (2001) Acute neuronal apoptosis in a rat model of multiple sclerosis. J Neurosci 21: 6214–6220. 11487644 10.1523/JNEUROSCI.21-16-06214.2001 PMC6763179

[59] O’Doherty C, Favorov A, Heggarty S, Graham C, Favorova O, et al (2009) Genetic polymorphisms, their allele combinations and IFN-beta treatment response in Irish multiple sclerosis patients. Pharmacogenomics 10: 1177–86. 19604093 10.2217/PGS.09.41 PMC2727921

